# The research progress of targeted therapy in acute myeloid leukemia based on bibliometric analysis

**DOI:** 10.3389/fonc.2022.957370

**Published:** 2022-09-02

**Authors:** Wanxue Huang, Gongrui Sun, Qi Wang, Zhiguo Long

**Affiliations:** ^1^ Department of Hematology, Fudan University Affiliated Pudong Medical Center, Shanghai, China; ^2^ Shanghai Key Laboratory of Regulatory Biology, Institute of Biomedical Sciences and School of Life Sciences, East China Normal University, Shanghai, China

**Keywords:** acute myeloid leukemia, aml, targeted therapy, CiteSpace, bibliometric analysis

Acute myeloid leukemia (AML) is a malignant proliferative clonal disease, characterized by a wide spectrum of molecular alterations. Although targeted therapy for AML has resulted in pronounced achievements in the past decade, clinical resistance caused by mutations in targeted oncogenes has been observed. A subset of patients with AML still requires the treatment of refractory and relapsed diseases. The field of targeted therapy in AML still presents many challenges. More studies are needed to overcome those obstacles. Through the CiteSpace tool, we can gain a systematic understanding of the current achievements in targeted therapy in AML, and we can predict hot research topics. Relevant publications from the Web of Science Core Collection were retrieved, and the acquired data were analyzed by CiteSpace to identify and predict trends and research hotspots in this field. We found that research on AML had focused on venetoclax resistance, novel therapies (such as targeting epigenetic modification), and *FLT3* mutation in the past decade. The clustering of keywords suggested interest in cell-targeted therapy (chimeric antigen receptor T cells and natural killer cells), signaling pathways (mTORC1), epigenetic therapies, and leukemic stem cells (LSCs). Drug resistance causing failure and relapse of treatment has raised increasing concern, and LSCs play a key role in relapsed AML, which represent an important direction for identifying targets for AML treatment in the future.

## Introduction

Acute myeloid leukemia (AML) is a heterogeneous disease caused by various gene mutations and cytogenetic abnormalities that affect the differentiation and proliferation of myeloid lineage cells (1). Advances in genomic investigations over the past 10 years have dramatically improved our understanding of mutated genes in AML and have allowed the tailoring of therapeutic strategies targeting mutated genes (2).

Several genes are recurrently mutated in AML, such as internal tandem duplication (ITD) of FMS-like tyrosine kinase 3 (*FLT3-ITD*), and mutations in isocitrate dehydrogenase (*IDH1/2*), nucleophosmin (*NPM1*), and/or CCAAT/enhancer binding protein alpha (*CEBPA*). As predictive biomarkers, these are indicators of prognosis and, in turn, can guide targeted therapeutic strategies, because patients harboring these specific mutations do not benefit from traditional chemotherapy (3, 4). Fortunately, tremendous progress has been made in deciphering the molecular pathogenesis of AML, enabling the development of target drugs, such as the introduction of small-molecule inhibitors of *FLT3*, *IDH1/IDH2*, and *BCL-2*. For example, the presence of ITDs in the *FLT3* receptor tyrosine kinase gene has long been known to confer a poor prognosis in patients with AML (5). The approval of multikinase *FLT3* inhibitor (*FLT3*i) midostaurin with induction therapy for newly diagnosed *FLT3*mut AML, and a more specific and potent *FLT3i* gilteritinib as monotherapy for relapsed/refractory (R/R) *FLT3*mut AML, has improved outcomes in patients with *FLT3-*mutated AML (4, 5).

However, targeted therapy in AML still faces challenges (6). Clinical resistance caused by mutations in the targeted oncogene has been observed (7–9). Furthermore, most cases of AML are without a targetable mutation (10). Many efforts have been made to explore new targets and develop the corresponding therapies. Numerous novel approaches that aim to achieve a new goal are currently being actively pursued.

To grasp the scope of research in this area, bibliometric analysis was applied to analyze the progress of targeted therapy in AML. Bibliometric analysis is a method used to analyze enormous amounts of heterogeneous literature. It can be applied to estimate the impact of research areas and to identify emerging trends (11). CiteSpace is a bibliometric tool, which aimed to analyze and visualize trends and patterns in the scientific literature, and presents the structure and distribution of current scientific knowledge (12). CiteSpace can be applied to visualize research frontiers, the knowledge base, and time spans, as well as the literature that has played a key role in research evolution. It focuses on finding critical points in the development of a field or a domain, especially intellectual turning points and pivotal points (13). The visual analytic tools applied in this review supplement traditional review, and survey articles and the findings are valuable for identifying critical developments from a vast number of published studies. In this review, we examine the evolution of targeted therapy in AML and provide a critical perspective on the clinical development of a variety of targeted treatments.

## Materials and methods

### Data sources and search strategy

The publications were obtained from the Web of Science Core Collection database (WoSCC). The strategy used during the search was Topic search #1 = (“acute myeloid leukemia” OR “AML”) AND Topic search #2 = (“targeted therapy*”), and then, the results were refined by [Document Types = (Articles or Review) Timespan: 2012-01-01 to 2021-12-31]. A total of 4,205 results were found. After data cleaning, duplicate publications were removed by CiteSpace v.5.8.R3; and ultimately, 4,150 total unique records were used in the final analysis. All records were then imported into CiteSpace v.5.8.R3.

### Algorithms and parameters in scientometric analysis

The time slices for analysis were set at 1 year, and the sources selected included all publication details—title, abstract, supplementary keywords and author keywords, selected keywords, author, institution, country for the node, selected g-index for the threshold, and the labels of clusters chosen by the log-likelihood ratio (LRR) test method algorithm—were used in the subsequent analysis. Pathfinder was selected for fine-tuning the setting to highlight key points. Betweenness centrality (BC) >0.1 was considered as pivot nodes. Modularity (Q) >0.3 was considered reasonable for the group. Silhouette >0.5 was considered homogeneity clusters. BC can be used to partially assess the influence of each node in the network (14). Usually, a node with a BC >0.1 is displayed as a purple ring, whose size is associated with the transformative potential of a scientific contribution (15). In addition, cluster analysis was used as another important approach to easily analyze knowledge networks in CiteSpace. More specifically, terms are classified according to their similarity and scored by specific algorithms, and then, the term with the highest score of each cluster is selected as the label of the cluster (16, 17).

### Other statistical analyses

The R programming language (version 4.1.0) was used for data visualization (packages ggplot2, tidyverse, ggthemes, ggsci, maps, patchwork, and RColorBrewer).

## Results

### Annual publishing and cite trends

**Figure 1:**
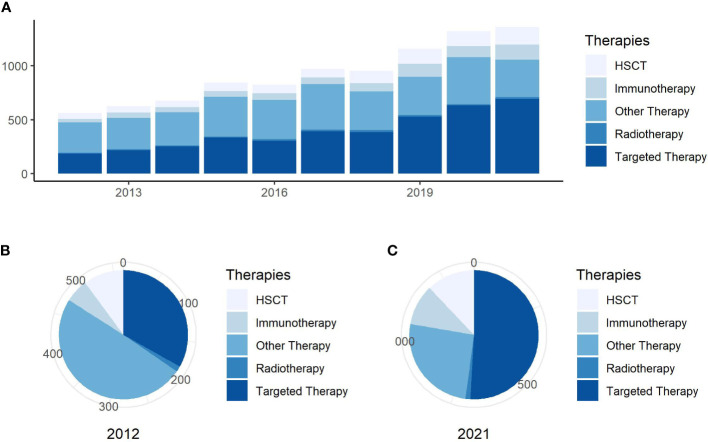
Annual publishing and proportion trends. **(A)** Trends in the number of publications per year on general research on AML treatment. **(B)** Proportion of publications about research for different AML treatment in 2012. **(C)** Proportion of publications about research into different AML treatment in 2021.

The annual number of published articles is a significant indicator for studying research trends in the field and reflects the pace of subject knowledge (18). We found a total of 129,587 citations for AML, with 83,287 articles cited, and an h-index of 142 in the field of targeted therapy in AML. The proportion studies investigating targeted therapies published annually in overall AML treatment are visually displayed in **Figure 1**. The number of targeted therapy documents in AML has increased annually between 2012 and 2021. Furthermore, the proportion of targeted therapy in total treatment has gradually increased, with the number of articles growing much faster.

### Country and institution analysis

**Figure 2:**
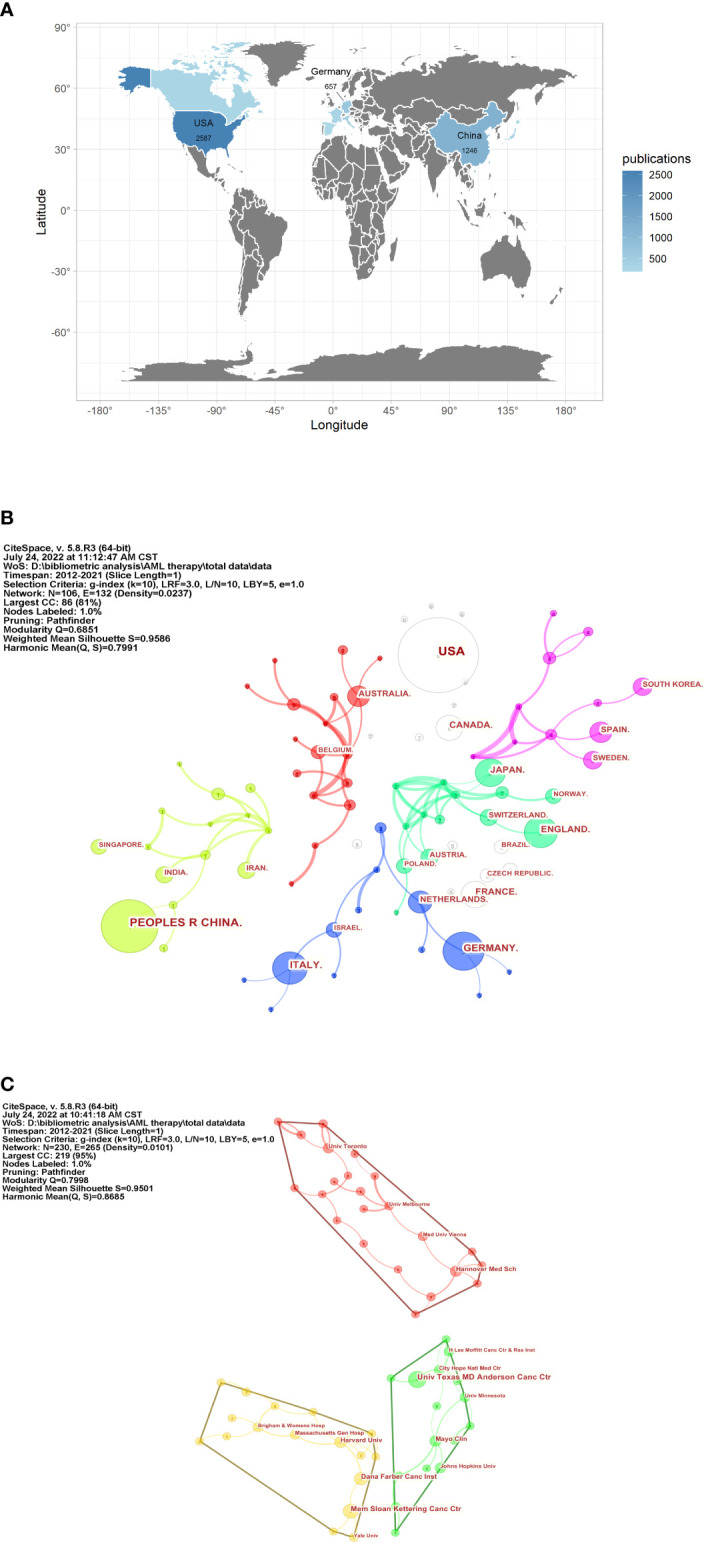
Country and institution analysis of targeted therapy in AML. **(A)** Top 10 countries for publications on the world map. **(B)** The network of countries for co-authored articles on targeted therapy in AML. Nodes represent publications from countries. The more frequently the publications of countries are cited, the larger is the size of the node. Links between nodes describe a co-occurrence or a co-citation between these nodes, and their thickness indicates the strength of these correlations: the thicker the line, the closer is the connection between them. The node color and link color indicate different clusters; the nodes that are of the same color belong to the same closely related cluster. **(C)** The network of institutions.

Visualized knowledge maps and institutional networks can provide information on the cooperative relationship between different research teams and countries (19). An analysis of the country distribution of these publications indicates that the United States, China, and Germany ranked the top three in the number of citations to articles. The United States (BC = 0.19) and Germany (BC = 0.14) were the countries that are most frequently associated with co-authored articles due to the highest BC (**Figures 2A, B**).

The network of institutions participating in publications is visualized in **Figure 2C**. The MD Anderson Cancer Center of the University of Texas, Memorial Sloan Kettering Cancer Center, Dana-Farber Cancer Institute, Harvard Medical School, and Harvard University ranked the top five institutions for the number of articles published. High BC values were obtained from the Dana-Farber Cancer Institute (BC = 0.12) and Harvard Medical School (BC = 0.10), which were also in different clusters.

### Co-author and categories analysis

**Figure 3:**
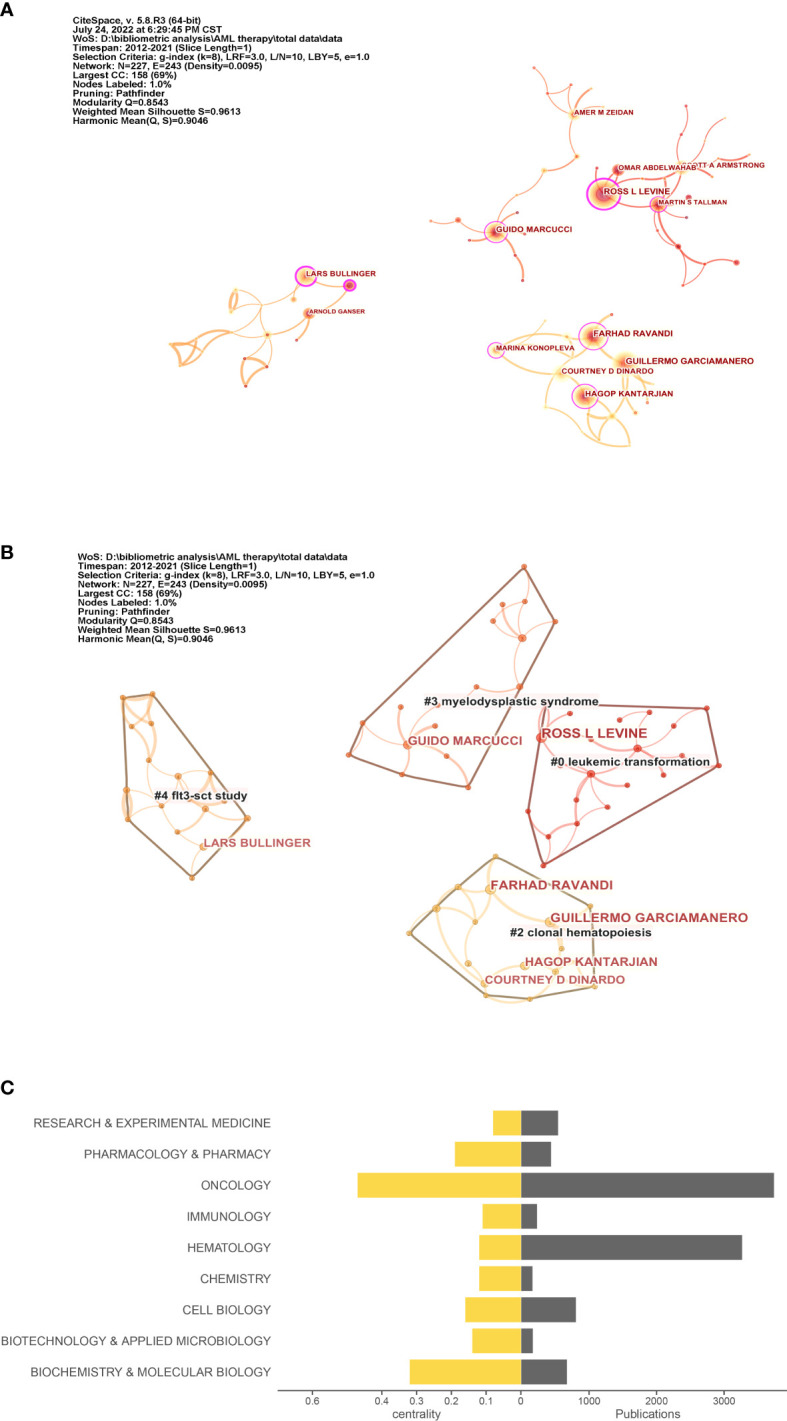
Co-author and category analysis. **(A)** Co-author network. The circle in **(A)** indicates the author; the larger the size of the circle, the higher the number of citations of the author. **(B)** Clusters of the co-author network. **(C)** Top 10 topics for publications. The right axis is the publication, and the left axis is the BC value.

BC measures the extent to which a node plays a bridging role or influence in a network. Specifically, BC measures the extent that the node falls within the shortest path between other pairs of nodes in a network. If the author plays a bridge that makes connections with other authors, then author’s BC value will high. The more an A depends on B to make connections with other people, the higher that B’s BC value (20). In particular, Lars Bullinger (BC = 0.29) and Ross L. Levine (BC = 0.22) had extensive contact with other researchers. Moreover, Ross L. Levine, Farhad Ravandi, and Guillermo Garcia-Manero were included the top three categories of citations (**Figure 3A**). In **Figure 3B**, cluster groups can be observed. The connection density between these groups was higher than that between the other nodes. The nodes within each group were uniform, and the number of publications and BC of co-occurring subject categories in AML are shown in **Figure 3C**.

### Co-keyword analysis

**Figure 4:**
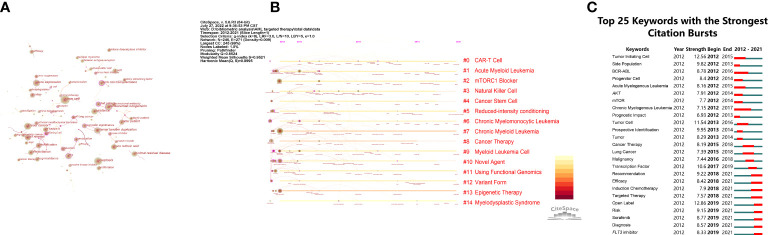
Co-occurrence and clustering of keywords. **(A)** Network of main keywords in publications. The circle in **(A)** indicates the keyword; the larger the size of the circle, the higher the frequency of the keyword. Purple circles represent the node of BC > 0.1: purple circles are thicker and the BC is higher. **(B)** Timeline view of keywords. Each horizontal line represents a cluster; the circular nodes on the line represent the top three keywords with the highest frequency of occurrence in this time slice. The timeline is shown at the top of the figure, and the year corresponding to the node is its publication time. The link between nodes represents the co-citation relationship. **(C)** The 25 keywords with the strongest citation bursts. The blue line denotes the time axis, whereas the red segment on the blue time axis shows the burst detection, indicating the start year, end year, and burst duration.

As a highly condensed summary of the paper’s content, keywords can be used to summarize the topics of the paper simply and directly. When the co-occurrence network of the keywords was dense, we applied Pruning functions to select important connections to highlight the key aspects of the network. The co-occurrence of targeted therapy keywords in AML and related research can be visualized in **Figure 4A**. **Figure 4B** shows the top 14 keyword clusters based on the LLR algorithm. This information, which consisted of highly cited and representative terms, in each group is summarized in **Table 1**. Burst detection is also a feature of CiteSpace, and a positive node after burst detection indicates a sharp change in its frequency of citations over a short period of time (15). Such nodes usually suggest a shift in a certain field of research and are indicated in red on the knowledge map. The identification of a research focus was based on the keyword co-occurrence network. The top 25 keywords with the strongest citation bursts relative to targeted therapy in AML in the last 10 years are shown in **Figure 4C**.

**Table 1:** The information of clusters about keyword co-citation analysis.

Clusters	Label (LLR)	Terms
0	CAR-T cell	CAR-T cell; multiple myeloma; robust antitumor potential; CD7-positive malignancies; nanobody exhibit
1	Acute myeloid leukemia	acute myeloid leukemia; *FLT3* inhibition; *FLT3* inhibitor; acute promyelocytic leukemia; possible strategies
2	mTORC1 blocker	mTORC1 blocker; Hedgehog pathway inhibition; other BCL-2 family member protein; mTORC cascade inhibitor; anti-leukemic role
3	Natural killer cell	natural killer cell; adoptive cell therapy; hematologic malignancies; promising therapeutic target; cellular therapy
4	Cancer stem cell	cancer stem cell; essential role; acute myeloid leukemia; myelodysplastic syndrome patient; cancer therapy
5	Reduced-intensity conditioning	reduced-intensity conditioning; acute myeloid leukemia; cancer stem cell; pediatric acute myeloid leukemia; allogeneic stem cell transplantation
6	Chronic myelomonocytic leukemia	chronic myelomonocytic leukemia; ITD-positive acute myeloid leukemia; *FLT3* inhibitor; mutant kinase; elderly high-risk myelodysplastic syndrome
7	Chronic myeloid leukemia	chronic myeloid leukemia; targeting mTOR; death knell; CD33-targeting drug; drug resistant lung cancer
8	Cancer therapy	acute myeloid leukemia; cancer stem cell; cancer therapy; emerging role; targeting cancer stem cell
9	Myeloid leukemia cell	myeloid leukemia cell; ultra-deep amplicon; monitoring therapy responses; leukemic subclone level; self-renewal activity
10	Novel agent	acute myeloid leukemia; cancer stem cell; novel agent; antibody-drug conjugate; refractory acute myeloid leukemia
11	Using functional genomics	acute myeloid leukemia; cancer stem cell; using functional genomics; complementary mechanism; next-generation cancer treatment
12	Variant form	variant form; chronic myeloid leukemia cell; stem cell population; lung adenocarcinoma cell; mitochondria-associated cysteine-rich protein augments tumorigenicity
13	Epigenetic therapy	epigenetic therapy; cancer stem cell; cancer therapy; aberrant microRNA expression; microRNA-143 target
14	Myelodysplastic syndrome	myelodysplastic syndrome; pediatric AML; regulatory T cell; treatment efficacy prediction; MDS progression

### Reference co-citation analysis

The identification of core literature in a certain field depends on the frequency of citations, and references with the highest cited frequency, namely, high-impact publications, are usually the focus of researchers. The top 10 cited references with the highest number of citations are shown in **Table 2**. The themes of references with the highest frequency of citations are divided into three parts: guidelines (diagnosis and management of 2017 ELN recommendations, and 2016 WHO classification revision from 2016 WHO); reviews of prognostic relevance of genomic classification and epigenetic landscapes (*DNMT3A* mutations) in AML; and targeted drugs for AML (midostaurin plus chemotherapy for *FLT3* mutation, and enasidenib for relapsed or refractory AML).

**Table 2:** The top 10 cited references with the highest cited frequency.

**Rank**	**Title**	**Citation Counts**	**Cluster ID**	**DOI**
1	Diagnosis and management of AML in adults: 2017 ELN recommendations from an international expert panel	470	0	10.1182/blood-2016-08-733196
2	Genomic classification and prognosis in acute myeloid leukemia	461	0	10.1056/NEJMoa1516192
3	The 2016 revision to the World Health Organization classification of myeloid neoplasms and acute leukemia	434	2	10.1182/blood-2016-03-643544
4	Genomic and epigenomic landscapes of adult *de novo* acute myeloid leukemia	420	2	10.1056/NEJMoa1301689
5	Acute myeloid leukemia	290	5	10.1056/NEJMra1406184
6	Prognostic relevance of integrated genetic profiling in acute myeloid leukemia	272	1	10.1056/NEJMoa1112304
7	Midostaurin plus chemotherapy for acute myeloid leukemia with a *FLT3* mutation	209	0	10.1056/NEJMoa1614359
8	Diagnosis and management of acute myeloid leukemia in adults: recommendations from an international expert panel, on behalf of the European LeukemiaNet	178	1	10.1182/blood-2009-07-235358
9	Enasidenib in mutant *IDH2* relapsed or refractory acute myeloid leukemia	171	0	10.1182/blood-2017-04-779405
10	*DNMT3A* mutations in acute myeloid leukemia	166	1	10.1056/NEJMoa1005143

**Figure 5:**
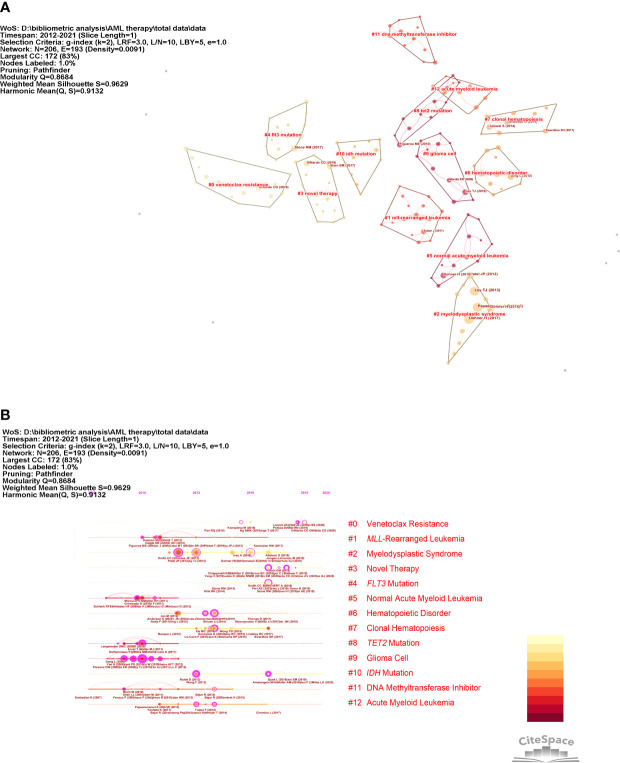
Co-citation network and timeline view of references. **(A)** Reference co-citation network. The circles in **Figure 4A** indicate references. **(B)** Timeline view of references. The circular nodes on the line represent the three most cited references in this time slice.

**Figures 5A, B** shows the co-citation network and the timeline view of reference sources on targeted therapy in AML and related studies. The timeline view is a visualization method that combines clustering and time-slicing techniques. Items are ranked according to their early or late appearance after clustering, displaying both the topic distribution in the field and illustrating the trends and interconnections of research topics over time. The references with the highest BC were Wang F 2013 (BC = 0.15), followed by Papaemmanuil E 2016 (BC = 0.12), Dombret H 2015 (BC = 0.10), Patel JP, 2012 (BC = 0.10), and Welch JS, 2016, (BC = 0.10), namely, these are the critical research authors driving research on the development of targeted therapy in AML. In **Figure 5B**, a straight line at the same horizontal position indicates all the references belonging to the group, with the group label located at the right-hand end of the line. Each cluster represents the fundamental knowledge of the underlying specialty.

As can be seen in **Figure 5B**, research on AML-targeted therapy focused on MLL-rearranged leukemia (cluster #1), TET2 mutations (cluster #9), and DNA methyltransferase cluster #11) from 2007 to 2013. Research on targeted therapy in AML shifted to venetoclax resistance (cluster #0), novel therapy (cluster #3), and *FLT3* mutations (cluster #4) from 2013 to 2021. The largest cluster focused on venetoclax resistance. The information, which consisted of highly cited and representative terms, in each group is shown in **Table 3**.

**Table 3:** The information of clusters about reference co-citation analysis.

Clusters	Label (LLR)	Terms
0	Venetoclax Resistance	venetoclax resistance; acute myeloid leukemia stem cell; BCL-2 inhibitor venetoclax; different face; intriguing clinical success
1	* MLL*-Rearranged Leukemia	*MLL*-rearranged leukemia; writers eraser; histone orthography; new epigenetic therapy; attractive target
2	Myelodysplastic Syndrome	myelodysplastic syndrome; acute myeloid leukemia; *de novo*; acute myeloid leukemia patient; *NPML*-mutated AML
3	Novel Therapy	novel therapy; targeting epigenetic modification; epigenetic therapy combination; new metabolic therapeutic target; microenvironment-derived metabolite
4	* FLT*3 Mutation	*FLT3* mutation; refractory acute myeloid leukemia; *FLT3* inhibitor; overcoming resistance; targeted therapy
5	Normal Acute Myeloid Leukemia	normal acute myeloid leukemia; intermediate-risk acute myeloid leukemia therapy; determining risk; personalizing therapy; treatment strategy
6	Hematopoietic Disorder	hematopoietic disorder; pre-leukemic phase; signaling pathway; targeting novel; resistant acute myeloid leukemia
7	Clonal Hematopoiesis	clonal hematopoiesis; therapy-related myeloid neoplasm; myelodysplastic syndrome; high-throughput sequencing; acute myeloid leukemia
8	* TET2* Mutation	*TET2* mutation; unfavorable overall survival; stem cell; AML study group; clinical analysis
9	Glioma Cell	glioma cell; *IDH* mutation; mutant *IDH 1*; isocitrate dehydrogenase mutation; pre-leukemic stem cell
10	* IDH* Mutation	*IDH* mutation; clonal architecture; evolutionary dynamics; *IDH 2* inhibition; cancer development
11	DNA Methyltransferase Inhibitor	myelodysplastic syndrome; DNA methyltransferase inhibitor; epigenetic therapy; hypomethylating agent; predicting response
12	Acute Myeloid Leukemia	myelodysplastic syndrome; acute myeloid leukemia; molecular testing; genetic mutation; molecular pathogenesis

## Discussion

### General information

Targeted therapy in AML has received increasing attention. In the past decade, the number of publications in this field has increased rapidly. Through the co-authored network analysis of countries and institutions, we found that the United States and Germany were the representative countries with the highest BC and were found in different clusters, surrounded by other countries. The MD Anderson Cancer Center of the University of Texas, the Memorial Sloan Kettering Cancer Center, the Dana-Farber Cancer Institute, and Harvard Medical School, and Harvard University ranked the top five in the number of articles published.

Ross L. Levine, Farhad Ravandi, and Guillermo Garcia-Manero were the top three most cited authors. We observed that Lars Bullinger and Ross L. Levine had extensive contact with other researchers. Farhad Ravandi and Guillermo Garcia-Manero collaborated closely and specialized in state-of-the-art treatments for leukemia at The University of Texas MD Anderson Cancer Center (21). The research field of the co-author could be determined from the clusters of label names. Related fields involved the following topics: leukemic transformation, clonal hematopoiesis (CH), myelodysplastic syndrome (MDS), and *FLT3* study. Of these, the year of CH clustering was the most recent. We also noticed that the following categories were involved in driving the development of the field: biochemistry and molecular biology, pharmacology and pharmacy, immunology, applied microbiology and biotechnology, and chemistry. There are various degrees of interrelatedness between targeted therapy in AML and different categories, namely, the field of targeted therapy in AML is innovative and interdisciplinary.

We can discover topic distribution in the field and trends of research topics over time. Through the co-citation timeline, we found that research on targeted therapy in AML had changed to venetoclax resistance (cluster #0), novel therapy (cluster #3), and *FLT3* mutation (cluster #4) over the past decade. The clustering of keywords focused on cell-targeted therapy [chimeric antigen receptor T cells (CAR-T) and natural kill cells], signaling pathways (mTORC1), epigenetic therapies, and leukemic stem cells (LSCs). Combined with clustering of keywords and cited references, it is obvious that LSCs appeared not only in clustering of keywords but also in clustering of cited references, such as the largest cluster representing venetoclax resistance.

### Research progress on targeted therapy in AML

#### Tyrosine kinase 3 (FLT3) inhibitor

The key term “*FLT3* inhibitor” was present in a cluster of co-references and in the keywords having the strongest citation burst, respectively. Up to 2017, the European Leukemia-Net (ELN) adjusted and updated cytogenetic and genomic mutations to the classification based on relative evidence. ELN categories were developed to correlate with genetic abnormalities having a clinical prognostic impact. The categories of these guidelines are divided into favorable, intermediate, and adverse. The adverse categories included *FLT3-ITD* with a high allelic ratio (22). *FLT3* mutations associated with an adverse prognosis have been identified in approximately one-third of patients with AML and represent an attractive therapeutic target.

The clinical development of *FLT3* inhibitors is one of the most active fields in AML (23). The standard first-line treatment for AML had not changed for more than 45 years (24). With the advent of *FLT3* inhibitors, the treatment armamentarium for *FLT3*-mutated AML has begun to expand. First-generation agents, such as midostaurin, sorafenib, and lestaurtinib, are broad-spectrum tyrosine kinase inhibitors. Midostaurin became the first targeted therapy approved by the United States Food and Drug Administration (FDA) for *FLT3*-mutated AML in 2017 (25, 26). In the international randomized phase III RATIFY trial, the multikinase inhibitor midostaurin significantly improved overall and event-free survival in patients aged 18 to 59 years of age with *FLT3*-mutated AML (27). Data from a phase II single-arm trial have provided evidence that midostaurin also improves the outcome of patients of 60 to 70 years of age with *FLT3*-ITD positive AML (28). The National Comprehensive Cancer Network Clinical Practice Guidelines recommend clinic trails for patients harboring molecular mutations. When a complete remission (CR) is observed, enrollment in a clinical trial is recommended for patients. Other recommendations include intermediate dose cytarabine and midostaurin for patients with *FLT3*-mutation–positive AML (28, 29). In addition, next-generation *FLT3* inhibitors, such as gilteritinib, quizartinib, and crenolanib, are more selective and have shown promising activity as single agents in early phase trials (30).

Although considerable progress has been made in the treatment of AML by targeting *FLT3*, many challenges still remain. Many resistance mechanisms have been identified with *FLT3* inhibitors, but not all have been fully elucidated. These resistance patterns render *FLT3*-targeted molecules ineffective in many ways, making them difficult to overcome. Furthermore, although several *FLT3* inhibitor studies have included maintenance therapy (31), additional studies are still needed to confirm these findings.

#### Epigenomic landscapes

Aberrations of DNA methylation also rank among the most frequent alterations observed in patients with AML. Recurrent somatic alterations in myeloid malignancies of key proteins involved in DNA methylation have highlighted the importance of epigenetic regulation of gene expression in the initiation and maintenance of various malignancies (32).

*DNMT3A* is one of several epigenetic modifiers identified as recurrently mutated in AML. *DNMT3A* mutations have also been identified in patients with MDSs (31) and myeloproliferative neoplasms (33) and are associated with a greater likelihood of progression to AML. In fact, in some studies, the same *DNMT3A* mutation as the antecedent hematologic disorder is identified in secondary AML, suggesting that these mutations may be an early event in malignant clonal evolution (34). These observations are further reinforced by recent findings demonstrating that recurrent *DNMT3A* mutations are frequently present in a pool of preleukemic clonal hematopoietic stem cells (HSCs) from which AML develops (35). These HSCs have a competitive multilineage repopulation advantage over wild-type HSCs and also have been demonstrated to persist after chemotherapy, thus acting as a reservoir for therapeutic resistance (36). Epigenetic alterations result in changes in gene expression in the absence of modifications of the relevant DNA sequence. Such alterations have the potential to promote stem cell renewal and alter progenitor cell differentiation (4, 37).

In other citations, *TP53* mutations and decitabine treatment have a high BC value in AML and MDS. With more and more researchers focusing on the role of DNA methylation in AML, decitabine (5-aza-2-deoxycytidine), a strong specific inhibitor of DNA methylation, is commonly used as a single agent to treat patients with MDS and older aged patients with AML (38). However, response rates are low. Extended treatment exposure to decitabine (administered on days 1 through 10 of the 28-day cycles instead of on days 1 through 5) shows an improved response rate (ranging from 40% to 64%). Patients with AML and MDS presenting cytogenetic abnormalities associated with unfavorable risk, TP53 mutations, or both had favorable clinical responses and robust (but incomplete) mutation clearance after receiving serial 10-day courses of decitabine. They achieved overall survival rates similar to those among patients with AML who had an intermediate-risk cytogenetic profile and who also received serial 10-day courses of decitabine (39).

Targeted epigenetic therapies are effective therapies in advanced preclinical and early clinical development (40). Isocitrate dehydrogenases (*IDHs*) are present in 15%–30% of AML patients (41). If mutations occur in *IDH1* and *IDH2*, then it would leads to gene hypermethylation, resulting in cellular proliferation, aberrant gene expression, and the inhibition of myeloid differentiation (42). It is reported that co-occurrence of *IDH* and *TET* methylcytosine dioxygenase 2 (*TET2*) mutations leads to DNA hypermethylation, contributing to leukemogenesis (41, 43, 44). *IDH1/2* inhibitor interrupts epigenetic changes. Recently (45), *IDH* inhibitors have been approved by the FDA: the *IDH1* inhibitor ivosidenib (AG-120) and the *IDH2* inhibitor enasidenib (AG-221) (43).

Advances in genomics, epigenetics, and drug discovery have led to the development of several potential novel therapeutic agents, many of which are being investigated in ongoing clinical trials. Additional studies will be necessary to determine how best to incorporate these novel agents into the routine clinical treatment of AML (10). The epigenetic therapies for AML might be one of the most important future treatment options (46, 47).

#### Mixed lineage leukemia–rearranged leukemia

Mixed lineage leukemia (*MLL*) family, also named the human *KMT2* family, due to the role of the first member *KMT2A* was found in this disease. Recent exome sequencing studies have revealed that the *KMT2* genes are among the most frequently mutated genes in many types of human cancers (48). Rearrangements in the *MLL* gene cause aggressive AML leukemias that follow an aggressive clinical course with poor response to conventional chemotherapy and frequent early relapse (49).

A new menin-*MLL* inhibitor (VTP-50469) appears to promote leukemia cell differentiation through direct effects on the *HOX* cofactor MEIS1 [*HOX* expression has been shown to be essential to maintain the leukemic phenotype of *MLL*r (50)]. The *MEIS1 HOX* cofactor plays a key role in maintaining a functional *HOX* network in AML (51)), paving the way for clinical trials (10). VTP-50469 not only induced loss of Meis1 expression and significant differentiation of *cytosolic NPM1(NPM1*c*)* leukemic cells but also prevented long-term engraftment and subsequent transformation of *NPM1*c-GMPs into leukemic cells after secondary transplantation in mice. Furthermore, the inhibitor was identified to suppress growth and induced differentiation in human *NPM1*c AML cells in patient-derived xenograft models (52).

In addition, there are other promising novel drugs: the DOT1L inhibitor pinometostat is activated in *KMT2A*-rearranged AML, KO-539, and SNDX-5613 (both are menin inhibitors) useful for *KMT2Ar* and *NPM1*-mutated AML (10). For example, in a phase I trial, KO-539 may be active in patients with AML. The agent induced CRs in two patients with relapsed/refractory disease and showed signs of activity in several patients (53).

The increasing availability of high-throughput genomic technologies in clinical settings allows a more accurate diagnosis of *MLL*-rearranged leukemia, which may provide an individual therapeutic strategy in time. The menin-*MLL* inhibitor may become a promising preclinical drug and worth investigating in the future, which will benefit patients with poor response to conventional chemotherapy and frequent early relapse.

### Research hotspots and focus

#### Leukemia stem cells

Recently, research has focused on LSCs in AML (54). Mounting evidence shows that LSCs are the key drivers of relapse in AML (55).

Relapse is a major problem in AML. Although chemotherapy can achieve CR in most patients with AML, approximately two-thirds of patients relapse within 18 months (56). The underlying reason for treatment failure is increasingly attributed to the presence of a drug-resistant subpopulation of AML cells, especially leukemia-initiating cells or LSCs (57). Most HSCs are usually in a state of quiescence. A subpopulation of AML cells is the same as HSCs, which means that LSCs also acquire quiescence. Quiescence is an important contributor to LSC drug resistance, as conventional chemotherapeutics target dividing cells (58).

The clonal representation of AML at the time of disease relapse reflects the continued evolution of LSC in many patients during remission. At relapse, minor clones present at diagnosis can emerge as dominant ones, or founder clones can re-emerge with new subclonal structures (59, 60). Even in rare cases of late relapse, residual LSCs from founder clones are the usual cause (61). In many reported cases, new mutations, particularly transversions, are present in relapsed clones (59, 60), suggesting that chemotherapy itself induces DNA damage that accelerates LSC clonal (62).

Treatment regimens targeting the quiescent LSC population or targeting niche-driven drug resistance are beginning to emerge. For example, combination drug treatments where the first treatment aims to activate quiescent LSCs or remove them from the quiescence driving niche, such as CXCR4 antagonists or E-selectin inhibitors that potentially block CD44 activation, combined with a cytotoxic drug, hold promise (63, 64). The differences between LSC and HSC quiescence are an important direction for the identification of targets for AML treatment in the future.

#### Clonal hematopoiesis

The number of gene mutations increases with age. Strong evidence in AML points to the origins of LSCs in preleukemic cells that arise through the sequential accumulation of somatic DNA mutations in HSCs (65, 66). Among those mutations, one may arise that confers a fitness advantage for a cell. When this process occurs in the hematopoietic system, a substantial proportion of circulating blood cells can derive from a single mutated stem cell, which is called “clonal hematopoiesis” or CH (56, 67).

Early mutations enhance or acquire self-renewal potential and differentiation impairment (68, 69), both of which can lead to variably expanded clonal populations of preleukemic HSCs in patients (60, 70). Late mutations in molecules within signaling pathways (for example, *FLT3*) promote proliferation, impose a full differentiation block, and drive the development of AML (71).

Previous research based on large samples found that CH is largely the result of mutations in fixed “early” mutational events: The genes that encode epigenetic modifiers, *DNMT3A* and *TET2*, are the two most common mutations; the third most commonly mutated gene was *ASXL1*, whereas mutations in splicing factors (*SF3B1*, *SRSF2*, *PRPF8*, and *U2AF1*) were also frequent (72).

Cancer-free individuals with somatic mutations in a cancer-associated variant (with a variant allele fraction equal or greater than 2%) should be considered to have CH of indeterminate potential (CHIP) (56). The most common mutations in CHIP are also recurrent drivers of AML. People with CHIP would be likely to develop AML, because they have the “first hit” needed for malignant transformation. Mutations in *DNMT3A*, *ASXL1*, *IDH1/2*, and *TET2* are often acquired at the early stage of the “two-hit hypothesis” (73–75). To refine the risk estimates for developing AML associated with CH, two groups have conducted studies with a large population having several years of follow-up (76, 77). The results indicated that individuals with antecedent CH had an approximately ~3- to 5-fold increased risk of developing AML in subsequent years (72).

The research into CH may be a promising field and worth focusing on. Such research could provide a theory for screening for people at especially high risk of transformation in the future. Additional studies are needed to understand and discover CHIP. Most importantly, it is necessary to find ways to reverse the pathogenic effects of CHIP. Ideally, drugs that can suppress mutant clones in the future should be identified, which could block malignant transformation in the “first hit” state to some extent.

#### Chimeric antigen receptor T cell

Recently, with the development of immunotherapy, CAR-T therapy targeting AML cells is undergoing active development (78). The treatment strategy for AML is shifting from being limited to two selections (cytotoxic chemotherapy followed by hematopoietic stem cell transplant or hypomethylating agent) to the availability of various novel target therapies (45).

After the great success in B cell malignancies, scientists turn to focus on CAR products that could target AML; however, there has been hindered by several obstacles. Numerous antigens are being investigated to find the ideal AML target, one that is expressed solely by the AML cell, including the LSCs, and that is a driver for AML proliferation. The majority of CAR T or NK cell antigens are cell surface antigens, which are commonly expressed by normal HSCs. Targeting intracellular antigens with CAR T or NK cells is a much more laborious task that involves expression of the intracellular antigen, or segments of the antigen, on the cell surface. FLT3, transmembrane receptor tyrosine kinase, has a vital role in maintaining normal HSC and progenitor cell function, including proliferation and differentiation. Wang et al. described potent *in vitro* cytotoxicity of FLT3 CAR Ts in AML cell lines, especially in cells harboring the FLT3-ITD mutation (79). Surprisingly, the growth of normal CD34^+^ HSC was not inhibited by the CARs. Administration of the anti-FLT3 CAR also prolonged survival of mice in a human FLT3^+^ AML xenograft mouse model.

### Further challenges

As we can observe, the largest cluster in the co-citation network of references is venetoclax resistance. Drug resistance causing treatment failure and relapse raises increasing concern. Despite the discovery of multiple molecular and targeted therapeutic targets and the ongoing development of various targeted therapies, there are still major challenges for resistance to targeted drugs.

Because of the genomic and/or epigenetic complexity of AML, with clonal heterogeneity and multiple lesions already present at diagnosis and subsequent further evolution throughout the course of the disease that often leads to the emergence of additional subclones with different resistance mechanisms, new targeted therapies are likely to have only moderate activity. When venetoclax is used as a single agent, therapeutic resistance inevitably evolves, typically within weeks or months.

The true anti-leukaemic potential of venetoclax was revealed only in combination with other agents, resulting in its approval in combination with hypomethylating agents or low-dose cytarabine, in elderly AML, but not as a monotherapy (80, 81). We have learned that the efficacy is markedly enhanced and might become evident only when the use of these agents is combined with other therapies, such as standard of care chemotherapies or HMA. Therefore, how to overcome drug resistance will become a new topic in the future.

### Limitations

There were some limitations in this study. First, our analysis was based on articles from the WoSCC database. Because of the restriction of the program, we only included Web of Science data. However, had more databases been included, a broader coverage of studies would have been provided. Second, because of the restriction of the program, the study interval was not extensive, with the extracted articles published from 2012 to 2021.

## Conclusions

This review has revealed that targeted therapy for AML has developed rapidly over the last decade. Through analysis of clusters (co-author, keywords, and cited references), we defined the topic distribution in the field and the trends in research topics over time. The research of targeted therapy in AML has shifted to venetoclax resistance, novel therapy (such as targeting epigenetic modification), and *FLT3* mutation during the past decade. The clustering of keywords focused on cell-targeted therapy (CAR-T and natural killer cells), signaling pathways (mTORC1), epigenomic and epigenetic targeted therapies, and LSCs.

The advances in FLT3 inhibitors and the discovery of epigenetic therapies (such as *IDH1*/*2* inhibitors) have led to the development of many potential novel therapeutic agents, many of which are being investigated in ongoing clinical trials. With the development of immunotherapy, CAR-T therapy targeting AML cells is rapidly developing. Therapy combination may give us possibilities for various treatments.

As we can observe, the largest cluster in the co-citation network of references was venetoclax resistance. Drug resistance that causes failure and relapse of treatment raises growing concern. LSCs play a key role in relapsed AML, which would be a challenge but important direction for identification of targets for AML treatment in future. Furthermore, as a pre-leukemic event, CH has also received attention in the field of targeted therapy in AML. Ideally, drugs that can suppress mutant clones in the future will be found, which could block malignant transformation in the “first hit” state to some extent.

Through bibliometric analysis, our study provides insight into the process of developing targeted therapy in AML and provides a perspective on the clinical development of a variety of precision treatment approaches. We hope that our study will provide researchers with a deeper understanding of AML pathogenesis and treatment.

## Author contributions

All authors listed have made a substantial, direct, and intellectual contribution to the work, and approved it for publication.

## Funding

The manuscript was supported by the Project of Key Medical Specialty and Treatment Center of Pudong Hospital of Fudan University (Grant No. zdzk2020-06).

## Acknowledgments

The authors are deeply indebted to ZL and GS for their support and effort in this study.

## Conflict of interest

The authors declare that the research was conducted in the absence of any commercial or financial relationships that could be construed as a potential conflict of interest.

## Publisher’s note

All claims expressed in this article are solely those of the authors and do not necessarily represent those of their affiliated organizations, or those of the publisher, the editors and the reviewers. Any product that may be evaluated in this article, or claim that may be made by its manufacturer, is not guaranteed or endorsed by the publisher.
